# Regulative Effect of Mir-205 on Osteogenic Differentiation of Bone Mesenchymal Stem Cells (BMSCs): Possible Role of SATB2/Runx2 and ERK/MAPK Pathway

**DOI:** 10.3390/ijms160510491

**Published:** 2015-05-07

**Authors:** Nan Hu, Chunzhen Feng, Yi Jiang, Qing Miao, Hongchen Liu

**Affiliations:** 1 Department of Stomatology, Chinese PLA General Hospital, Beijing 100853, China; E-Mails: sphm1987@fmmu.edu.cn (N.H.); sunjiy@fmmu.edu.cn (C.F.); bilinlin-05@fmmu.edu.cn (Y.J.); 2 Department of Pharmacy, 401 Military Hospital, Qingdao 266071, China

**Keywords:** mir-205, bone mesenchymal stem cells (BMSCs), special AT-rich sequence-binding protein 2 (SATB2), runt-related transcription factor 2 (Runx2), osteogenic differentiation, mitogen activated protein kinase (MAPK)

## Abstract

Bone mesenchymal stem cells (BMSCs) have multiple potentials to differentiate into osteoblasts and adipocytes, and methods to enhance their osteogenic differentiation are gaining increasing attention. MicroRNAs are critical regulation factors during the process of the osteogenic induction in BMSCs, and mir-205 has been substantiated to be involved in the osteogenic process, but the underlying mechanisms remain unclear. The purpose of this article is to investigate the role of mir-205 in the osteogenic differentiation of BMSCs. We found that mir-205 expression was down-regulated in a time-dependent manner during BMSC osteo-induction. Inhibition of mir-205 enhanced osteogenic abilities by up-regulating bone sialoprotein (BSP) and osteopontin (OPN) protein levels and increasing alkaline phosphatase (ALP) activity and osteocalcin secretion. Furthermore, we found that mir-205 could regulate protein expression of special AT-rich sequence-binding protein 2 (SATB2) and runt-related transcription factor 2 (Runx2), and over-expression of SATB2 activated Runx2 and reversed the negative effects of mir-205 on osteoblastic differentiation. Furthermore, we examined the extracellular signal-regulated kinase (ERK) and p38 mitogen-activated protein kinase (p38 MAPK) pathways during osteogenic induction and our data indicates that mir-205 might exert negative functions on the osteogenic differentiation in BMSCs at least partly via altering phosphorylation of ERK and p38 MAPK. These results shed new light on the molecular mechanisms of microRNAs in governing differentiation of BMSCs.

## 1. Introduction

Bone mesenchymal stem cells (BMSCs), isolated from the bone marrow by Friedenstein *et al.* [1], have multiple differentiation potentials. Numerous studies have shown that BMSCs can differentiate into osteoblasts, chondrocytes and adipocytes [2]. In a separate study, BMSCs even substitute for neural progenitor cells (NPCs) in restorative therapy for stroke [3]. BMSCs can differentiate into osteoblasts, which makes them a suitable cell type in bone repair and tissue engineering [4,5,6,7]. The osteogenic differentiation of BMSCs can be regulated by multiple signaling molecules and transcriptional regulators, and how to enhance the osteogenic differentiation of BMSCs has gained increasing attention in recent years.

MicroRNAs (miRNA) are small RNA molecules that bind to the non-coding region of mRNA and regulate mRNA activity by inducing mRNA degradation or suppressing mRNA activity [8,9]. MicroRNAs are involved in many progress, including cell proliferation, differentiation and death [10]. To date, several studies have substantiated that some miRNAs, such as mir-31, -34c, -204, -338-3p and so on [11,12,13], are involved in regulating the differentiation of BMSCs [14,15]. Mir-205 was previously known as a tumor suppressor and played an important role in tumor cell proliferation and migration [16]. However, recent studies showed that mir-205 was down-regulated during the induction of osteogenic differentiation in vascular smooth muscle cells [11]. However, the detailed mechanisms of mir-205 in regulating osteogenic differentiation remain unclear. Further, the role of mir-205 in BMSCs has not been characterized.

Special AT-rich sequence-binding protein 2 (SATB2) is a member of the family of special AT-rich sequence-binding proteins that binds to nuclear matrix-attachment regions (MARs) [17]. SATB2 plays a critical role in osteoblast differentiation. Studies have showed that SATB2 could regulate bone sialoprotein (BSP) and osteocalcin (OCN) expression by enhancing the activity of runt-related transcription factor 2 (Runx2) and activating transcription factor 4 (ATF4) [18]. Recently, SATB2 has been identified as a novel marker of osteogenic differentiation [19]. It was reported that mir-205 regulated osteoblast differentiation and formation by directly targeting Runx2, and whether mir-205 can regulate SATB2 has not yet been explored.

In this article, to investigate the regulative mechanism of mir-205 in osteogenic differentiation, we firstly detected the expression of mir-205 in BMSCs culture. Mir-205 inhibitor and mimics were employed to examine the effects of mir-205 on BMSC osteo-differentiation. Bioinformatics analysis and plasmid construction were used to illustrate the influence of mir-205 on STAB2 and Runx2. Our data showed that mir-205 negatively regulated osteogenic differentiation in BMSCs, and the regulative mechanisms might be mediated by STAB2 and Runx2 expression via the extracellular signal-regulated kinase (ERK) and p38 mitogen-activated protein kinase (p38 MAPK) pathways.

## 2. Results

### 2.1. Mir-205 Expression during the Process of Osteogenic Differentiation in Bone Mesenchymal Stem Cells (BMSCs)

**Figure 1:**
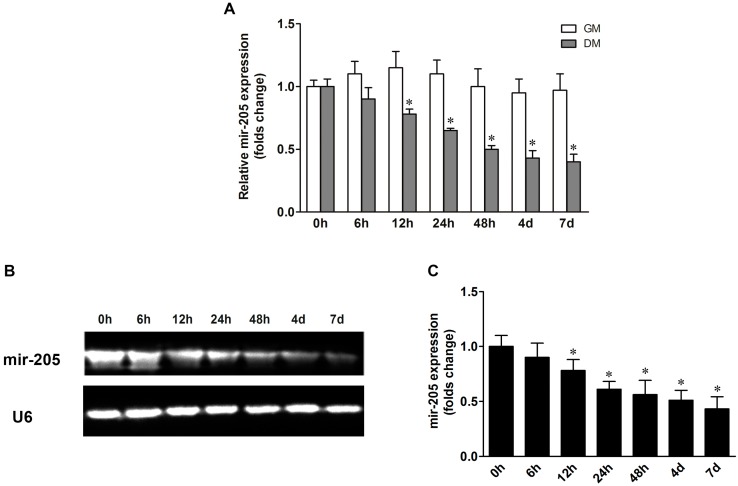
Mir-205 expression during the process of osteogenic differentiation in bone mesenchymal stem cells (BMSCs). BMSCs were treated with growth medium (GM) or differentiation medium (DM) for seven days to investigate the change in mir-205 levels. (**A**) Mir-205 expression was decreased along a temporal axis in BMSCs; (**B**,**C**) Northern blot bands data show that mir-205 was down-regulated in a time-dependent manner during osteogenic differentiation, and small nuclear RNA (U6) was used as control. Data are depicted as mean ± SD, * *p* < 0.05 *vs.* 0 h.

The qRT-PCR results showed that mir-205 expression was reduced in a time-dependent manner during the process of BMSCs osteogenic differentiation (Figure 1A). The mir-205 expression in BMSCs was decreased after treatment with differentiation medium (DM) from 12 h to 7 days. We also detected the mir-205 level in BMSCs culture with growth medium (GM), and no significant change was noted. To further confirm the mir-205 expression in BMSC osteogenic induction, northern blot analysis was conducted and the results were in good agreement with qRT-PCR results (Figure 1B,C). These data indicate that mir-205 is down-regulated during the process of osteogenic differentiation in BMSCs.

### 2.2. Inhibition of Mir-205 Enhances Osteogenic Differentiation

**Figure 2:**
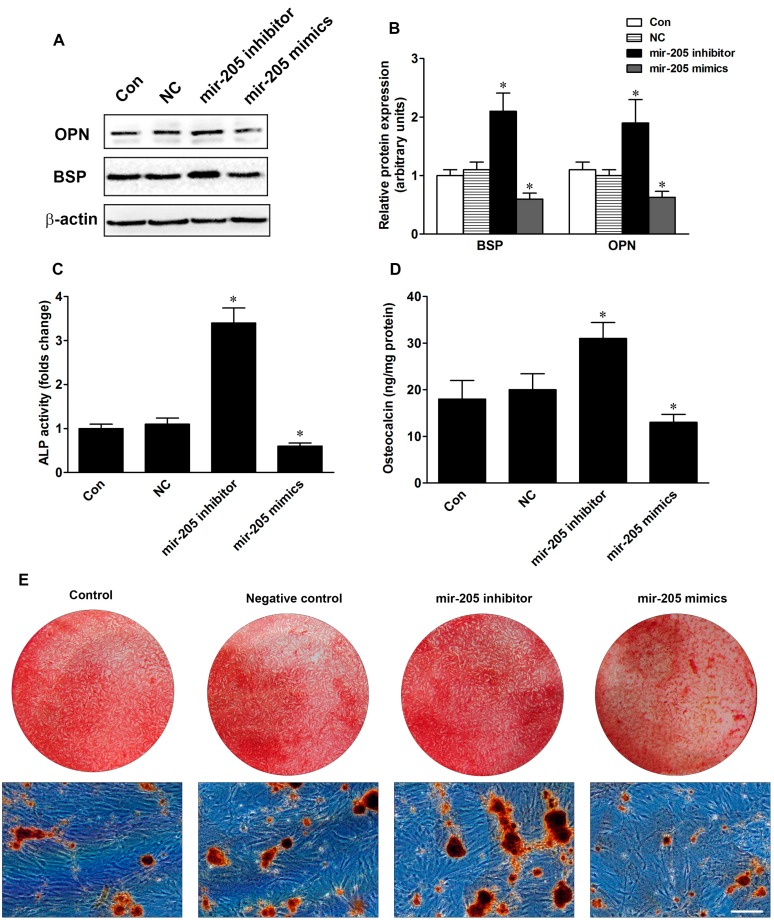

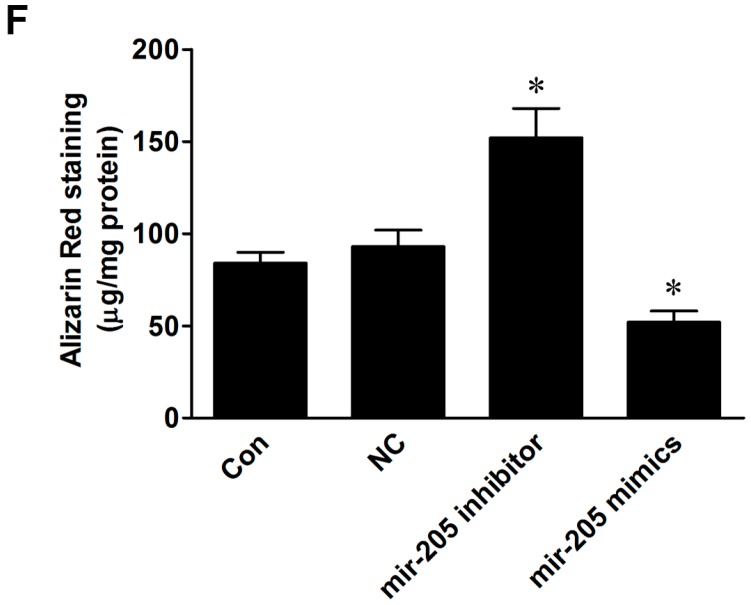
Effects of mir-205 on the osteogenic differentiation in BMSCs. (**A**,**B**) Bone sialoprotein (BSP) and osteopontin (OPN) protein expression after treatment with negative control (NC), mir-205 inhibitor or mir-205 mimics; (**C**) Alkaline phosphatase (ALP) activity was determined by ALP assay and expressed relative to total cellular protein; (**D**) Osteocalcin levels were measured by enzyme-linked immuno sorbent assay (ELISA) and expressed relative to total cellular protein; (**E**) Alizarin red S (ARS) staining images were shown at day 14. Scale bar = 200 μm; (**F**) Semi-quantity analysis of ARS staining. Data are depicted as mean ± SD, * *p* <0.05 *vs.* NC.

To investigate the role of mir-205 in the osteogenic differentiation of BMSCs, mir-205 inhibitor and mir-205 mimics were employed to change the expression of mir-205. Protein levels of bone sialoprotein (BSP) and osteocalcin (OCN) were examined in BMSCs cultured with DM after transfection for 48 h. As shown in Figure 2, BSP and OPN protein levels were significantly inhibited in BMSCs in the mir-205 mimic group, and the protein levels were ~40% and ~48%, respectively, of negative control (NC) (Figure 2B). Inhibition of mir-205 increased protein expression of BSP and OPN, and the folds of protein levels were 2.1 and 1.9 compared to the NC group. Furthermore, alkaline phosphatase (ALP) activity and OCN secretion levels were increased in mir-205 down-regulated BMSC cells, and reduced in mir-205 up-regulated BMSC cells (Figure 2C,D). Figure 2E,F showed the mineralization in BMSCs at day 14 after treatment with different vectors for seven days. Inhibiting mir-205 dramatically increased the mineralization effects compared with NC, but over-expression of mir-205 diminished the mineralization effects. Our results suggest that mir-205 negatively regulates osteogenic differentiation in BMSCs.

### 2.3. Special AT-Rich Sequence-Binding Protein 2 (SATB2) and Runt-Related Transcription Factor 2 (Runx2) Regulate Mir-205 Expression in BMSCs

**Figure 3:**
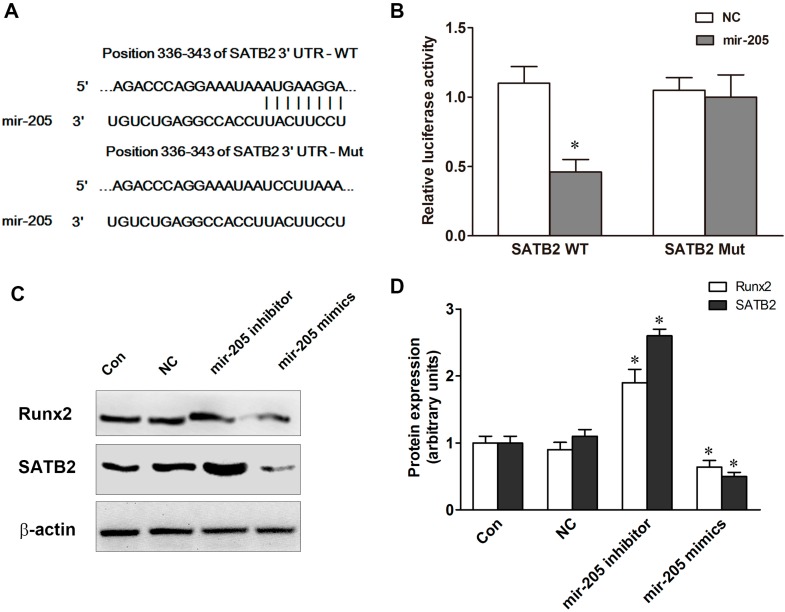
Special AT-rich sequence-binding protein 2 (SATB2) are targets of mir-205. (**A**) Schematic illustration of the predicted miR-205-binding sites in the 3'-untranslated regions (UTRs) of SATB2; (**B**) Luciferase reporter assays. BMSCs were co-transfected with luciferase reporter plasmids containing the wild-type (WT) or mutant (Mut) miR-205 target sites in the SATB2 or NC; (**C**,**D**) SATB2 and runt-related transcription factor 2 (Runx2) protein expression after treatment with NC, mir-205 inhibitor or mir-205 mimics. Data are depicted as mean ± SD, * *p* < 0.05 *vs.* NC.

Bioinformatics analysis showed that mir-205 was predicted as a potential miRNA binding motif on the SATB2 3'-untranslated region (UTR) (Figure 3A). To confirm the interaction between SATB2 and mir-205, BMSCs were transfected with mir-205 mimics and luciferase reporter constructs containing the wild-type (WT) or mutant (Mut) mir-205 target sites in the SATB2 3'-UTR. We found that up-regulation of miR-205 dramatically decreased the luciferase activity of the WT-SATB2-3'-UTRs of SATB2 and the luciferase activity was ~46% compared to NC (Figure 3A,B). Notably, no significant effect was found on the Mut-SATB2-3'-UTRs. To investigate the role of mir-205 on the expression of SATB2, mir-205 inhibitor or mir-205 mimics were transfected into BMSCs with DM, and western blot results showed that the protein level of SATB2 was reduced after treatment with mir-205 mimics and increased with mir-205 inhibitor (Figure 3C,D). Runx2 has been identified as a direct target of mir-205 [20,21]. In our studies, we also found that Runx2 protein level was increased almost two-fold in the mir-205 inhibitor group and decreased by ~50% in the mir-205 mimics group (Figure 3C,D). Our data suggests that mir-205 is involved in the regulation of SATB2 and Runx2 expression.

### 2.4. SATB2 Regulates Mir-205-Mediated Osteoblastic Differentiation in BMSCs

**Figure 4:**
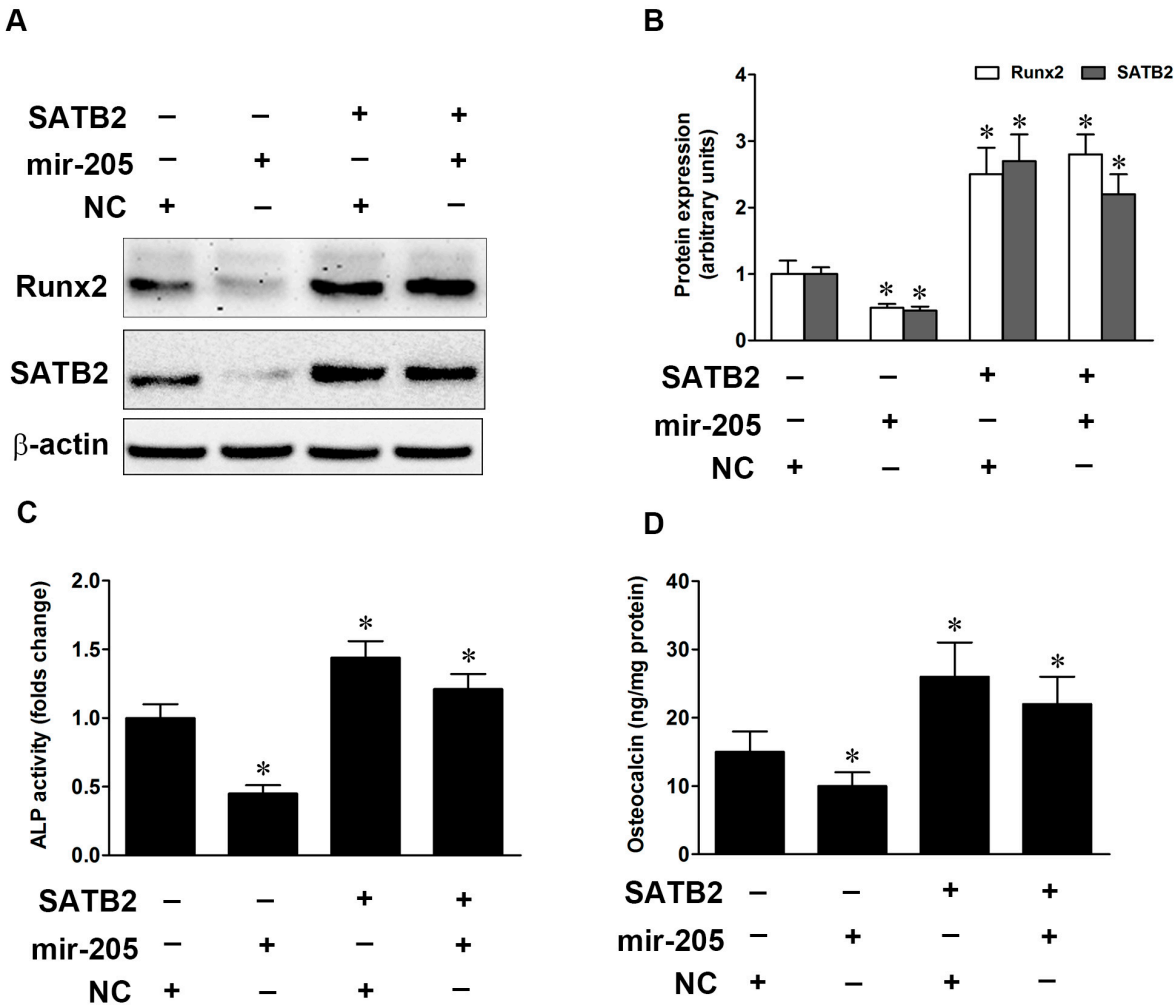
SATB2 activates Runx2 expression and regulates mir-205-mediated osteogenic differentiation. (**A**,**B**) SATB2 and Runx2 protein expression after transfected with SATB2 over-expression plasmid in mir-205 over-expression BMSCs; (**C**) ALP activity was determined by ALP assay and expressed relative to total cellular protein; (**D**) Osteocalcin levels were measured by ELISA and expressed relative to total cellular protein. Data are depicted as mean ± SD, * *p* < 0.05 *vs.* NC.

To evaluate whether the change in SATB2 expression mediated osteogenic differentiation in BMSCs, pEGFP-N1 plasmids were used to construct STAB2 over-expression vectors, and western blot data showed that SATB2 protein expression was significantly up-regulated in BMSCs and mir-205 mimics failed to alter the expression of SATB2 (Figure 4A,B). Previous studies showed that SATB2 could enhance the activity of Runx2, so we also detected the expression of Runx2 in SATB2-over-expressing BMSCs. Our results indicated that up-regulation of SATB2 increased the protein expression of Runx2. Next, we detected the ALP activity and OCN levels, and overexpression of SATB2 increased ALP activity and OCN levels with or without mir-205 mimics (Figure 4C,D). Our data established that SATB2 could regulate mir-205-mediated osteoblastic differentiation in BMSCs.

### 2.5. Inhibition of Mir-205 Increases the Phosphorylation of Extracellular Signal-Regulated Kinase (ERK) and p38 Mitogen-Activated Protein Kinase (MAPK) in BMSCs

**Figure 5:**
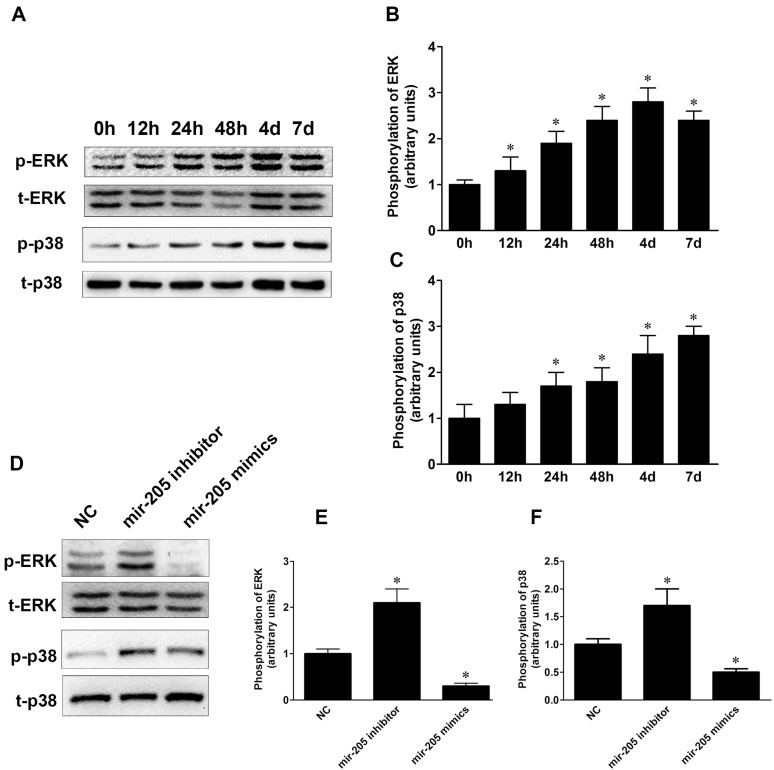
Phosphorylation of extracellular signal-regulated kinase (ERK) and p38 mitogen-activated protein kinase (MAPK) during the osteogenic differentiation of BMSCs. (**A**–**C**) Phosphorylation of ERK and p38 MAPK during the osteogenic differentiation. * *p* < 0.05 *vs.* 0 h; (**D**–**F**) BMSCs were treatment with DM and western blot analysis was used to verify phosphorylation of ERK and p38 MAPK after transfected with NC, mir-205 inhibitor or mimics. Data are depicted as mean ± SD, * *p* < 0.05 *vs.* NC.

Extracellular signal-regulated kinase (ERK) and mitogen-activated protein kinase (MAPK) signaling pathways have widely recognized roles in the osteogenic differentiation of BMSCs [22]. To investigate whether mir-205 regulated osteogenic differentiation via ERK/p38 MAPK pathways in BMSCs, western blot experiments were employed to detect the phosphorylation of ERK and p38. As shown in Figure 5A–C, BMSCs were cultured in DM, and phosphorylation of ERK and p38 increased in a time-dependent manner during the process of osteogenic differentiation. After transfection with NC, mir-205 inhibitor or mimics for 48 h, we found that inhibition of mir-205 increased phosphorylation of ERK and p38 MAPK, while overexpression of mir-205 inhibited activity of ERK and p38 MAPK during osteogenic induction (Figure 5D–F). Our results implied that mir-205 might negatively regulate osteogenic differentiation via ERK/MPAK signaling.

## 3. Discussion

The present study provides the detailed observations regarding the effects of mir-205 on osteogenic differentiation and possible mechanisms in BMSCs. Mir-205 negatively regulates osteoblastic differentiation via inhibiting the expression of Runx2 and SATB2, and the possible mechanisms may be mediated by the ERK and p38 MAPK pathways. Taken together, our results shed new light on the molecular mechanisms of microRNAs in governing BMSCs differentiation.

In recent years, great attention has been paid to deciphering the mechanisms of microRNA in regulating differentiation of BMSCs. Mir-31 was reported to play an important role in regulating the osteogenic differentiation of BMSCs [12]. Inhibiting mir-31 expression could increase ALP activity and mineralization in BMSC cultures, and over-expression of mir-31 reduced the expression of OPN, BSP and OCN [23,24]. Mir-548d-5p suppressed the dexamethasone-induced adipogenic differentiation by down-regulation of PPARγ, and increased mRNA and protein levels of OCN, Runx2 and ALP activity in BMSCs [25]. Mir-205 was previously reported as a tumor suppressor for numerous cancers, while studies have shown that mir-205 may be a key mediator in osteoblast differentiation. Bioinformatics information revealed that mir-205 could bind with many osteogenesis-related mRNAs, which implicated that the function of mir-205 was more complex than expected. Previous studies have reported that Runx2 and Smad1 were direct targets of mir-205 in vascular smooth muscle cells [21]. In a separate study, mir-205 expression could displace the TGF-β/Smad1 balance by targeting Smad1 and Smad4 [26]. In our experiments, we firstly detected the expression of mir-205 in BMSCs with proliferation medium and differentiation medium, and the results showed that mir-205 expression changed slightly in proliferative status, but decreased significantly in osteogenic differentiation of BMSCs (Figure 1). To confirm the effect of mir-205 on BMSCs, we altered the expression of mir-205 and found that inhibiting mir-205 enhanced the process of bone formation in BMSCs (Figure 2), which suggested that mir-205 negatively regulated osteogenic differentiation. A recent study showed that mir-205 negatively regulated the osteogenic differentiation in human aortic smooth muscle cells (HASMCs) [21]. Our results were in good agreement with previous studies.

Runx2 is a critical regulator of the osteogenic lineage, and its epigenetic functions modulate expression of bone-related genes [27]. Previous studies have identified that Runx2 is a target gene of mir-205 [11]. Mir-205 negatively regulated the osteogenic differentiation of HASMCs partially via targeting Runx2 and Smad1 [21]. Our data were in good agreement with previous studies that mir-205 could influence the expression of Runx2 (Figure 3), and we also found SATB2 was a direct target of miRNA. SATB2, as a member of the special family of AT-rich binding transcription factors, plays a pivotal role in craniofacial patterning and osteoblast differentiation [28,29]. SATB2 can binds to nuclear matrix-attachment regions (MARs) and activates Runx2-dependent osteoblast differentiation. It also can represses homeobox a2 (Hoxa2) expression in osteoblasts in a MAR-dependent manner, which suggests SATB2 has multiple inputs into transcriptional control of osteoblast differentiation [30]. Recently, several studies indicated that miRNAs mediated SATB2-induced osteogenic differentiation [31,32,33]. Mir-27a was significantly up-regulated in SATB2-over-expression cells and over-expression of mir-27a dramatically inhibited osteogenesis and repressed BMP and Smad9 expression [34]. We used bioinformatics analysis and found that mir-205 was predicted as a potential miRNA binding motifs on the SATB2 3'-UTR and luciferase activity experiment was used to confirm the relationship between mir-205 and SATB2 (Figure 3A,B). As expected, SATB2 protein expression was decreased in mir-205-over-expression BMSCs but increased in mir-205-down-expression BMSCs (Figure 3). Next, we overexpressed SATB2 in mir-205-over-expression BMSCs, and found that the osteogenic functions were restored from the negative effects of mir-205 (Figure 4). Notably, we found the protein expression of Runx2 was also increased, which suggested that up-regulation of SATB2 increased osteogenic differentiation probably via activating the expression of Runx2. Taken together, our results suggested that mir-205 induced decline in osteogenic differentiation of BMSCs via Runx2 and SATB2, and overexpressed SATB2 reversed the effect of mir-205 on the osteo-induction.

Previous studies have indicated that many signal transduction pathways, such as TGF-β, MAPK, Wnt and VEGF pathway, may be involved in the osteogenic differentiation of BMSCs [34]. The correlation between Runx2 and ERK/p38 signaling has been proved previously [35,36]. Runx2 is physiologically regulated by several cell-signaling pathways, including MAPK, TGFβ/BMP2, and Wnt [36]. Another study showed that the MAPK signaling pathway might participate in SATB2-induced osteogenic differentiation in BMSCs [34]. Previous studies showed that mir-205 possessed anti-angiogenic functions via impairing TGF-β pathway by targeting Smad1 and Smad4 in human umbilical vein endothelial cells (HUVECs) [26]. In a separate study, mir-205 induced differentiation of 3T3-L1 preadipocyte cells by targeting glycogen synthase kinase 3 beta (GSK-3β) [37]. However, the correlation between mir-205 and the ERK/p38 pathways has not yet been clarified. In our research, we observed that altering the expression of mir-205 during BMSC osteo-induction process could significantly influence the expression of SATB2 and Runx2, as well as change the phosphorylation of ERK and p38 (Figure 5). Therefore, our results suggested that the ERK and p38 MAPK pathways might take part in the regulation of mir-205 on BMSC osteoblastic differentiation. Further detailed research needs to be done to confirm the relationship between mir-205 and MAPK signaling.

## 4. Experimental Section

### 4.1. Cell Culture and Treatments

Primary culture of BMSCs were cultured from adult female Sprague Dawley rats as previously described [38]. All procedures were approved by the Animal Research Committee of Chinese PLA General Hospital, Beijing, China. Rats were sacrificed by cervical dislocation and bilateral femurs were dissected under aseptic conditions. Then, metaphyses from both ends were resected and bone marrow (BM) cells were collected by flushing the diaphysis with 10 mL of α-minimal essential medium (α-MEM; GIBCO Life Technology, Gaithersburg, MD, USA) with 10% heat-inactivated fetal bovine serum (FBS; GIBCO Life Technology, Gaithersburg, MD, USA), 100 U/mL penicillin and 100 μg/mL streptomycin. Cells were collected and seeded onto a 10 cm diameter plate at a density of 5 × 10^5^/mL, and incubated in 5% CO_2_ at 37 °C. The characteristics of BMSCs were validated using flow cytometry as previously described [38]. Briefly, cells were incubated with CD29 following a fluorescein-5-isothiocyanate (FITC)-conjugated secondary antibody, phycoerythrin (PE)-conjugated monoclonal antibody against CD90, FITC-conjugated monoclonal antibody against CD11b and PE-Cy5-conjugated monoclonal antibody against CD45. All incubations with antibodies were performed for 30 min. After two washes, cells were pelleted and resuspended in flow cytometry buffer containing 2% paraformaldehyde for 20 min, and data were acquired and analyzed on FACSCalibur with CellQuest software. For proliferation assays, growth medium (GM) consisted of α-minimum essential medium supplemented with 10% fetal bovine serum, 100 U/mL penicillin and 100 μg/mL streptomycin. For osteogenic induction, differentiation medium (DM) consisted of GM supplemented with 10 nM dexamethasone (Sigma, St. Louis, MO, USA), 10 mM β-glycerol phosphate (Sigma, St. Louis, MO, USA), and 50 mg/mL l-ascorbic acid. The medium was changed twice every week.

### 4.2. Alkaline Phosphatase (ALP) Activity Assay and Osteocalcin (OCN) Secretion

ALP activity was determined by utilizing the conversion of a colorless *p*-nitrophenyl phosphate (pNPP, Sigma, St. Louis, MO, USA) to a colored *p*-nitrophenol. In brief, after two washes with PBS, cells were scraped into a solution containing 20 mM Tris-HCl (pH 8.0), 150 mM NaCl, 2% Triton X-100, 0.02% NaN_3_, and 1 μg/mL aprotinin. Lysates were homogenized and ALP activity was determined at 405 nm using pNPP as the substrate at 37 °C. Results were normalized to total cellular protein contents. Osteocalcin secretion into the culture medium was measured by using a specific radioimmunoassay kit (DiaSorin Corp., Stillwater, MN, USA) according to the manufacturer’s instructions. Protein expression was measured by the Bradford protein assay. All experiments were conducted in triplicate.

### 4.3. Northern Blot Analysis

Total RNA was extracted from cell layers using a Trizol reagent (Sigma, St. Louis, MO, USA) and quantified with a NanoDrop ND-100 Spectrophotometer (NanoDrop Technologies, Wilmington, DE, USA). For quantification of miR-205, reverse transcription and polymerase chain reaction were performed using the TaqMan MicroRNA Reverse Transcription Kit and TaqMan miRNA assay (Applied Biosystems, Foster City, CA, USA). The probes were 5'-TCCTTCATTCCACCGGAGTCTG-3' for mir-205 and 5'-ATATGGAACGCTTCACGAATT-3' for U6. Thirty μg of total RNA samples were run on 12% acrylamide denaturing gels and then transferred to a Hybond-N^+^ nylon membrane by electrophoresis using a semi-dry transfer cell (Bio-Rad, Hercules, CA USA). Hybridization was performed according to a standard protocol.

### 4.4. Quantitative Real-Time (qRT)-PCR

RNA extraction was performed as noted above. Quantitative real-time PCR was performed with SYBR Premix Ex TaqTM Kit (Takara, Mountain View, CA, USA) using the applied Biosystem 7500 Fast Real-time PCR System (Applied Biosystem, Madrid, Spain). For qRT-PCR, primers for the indicated genes and GAPDH were designed with the aid of Clone Manager software. The sequences for each primer used herein are GAPDH (sense: 5'-GGCACAGTCAAGGCTGAGAATG-3'; antisense: 5'-ATGGTGGTGAAGACGCCAGTA-3'), BSP (sense: 5'-GATAGTTCGGAGGAGGAGGG-3'; antisense: 5'-CTAACTCCAACTTTCCAGCGT-3'), OCN (sense: 5'-GGAGGGCAGTAAGGTGGTGAA-3' antisense: 5'-GAAGCCAATGTGGTCCGCTA-3'), SATB2 (sense: 5'-GAAGGAGGAAAGAGAAAGGAAGAC-3' antisense: 5'-TCATTTATCTCGTGGGTCTTCC-3'). The sequences of U6 and mir-205 are described as noted above, and the universal reverse primer for miRNA qRT-PCR was obtained from One Step PrimeScript^®^ miRNA cDNA Synthesis Kit (Takara). The PCR was performed in strips, each sample examined in triplicate and a no-template control was included for each amplification. In order to verify the specificity of the amplification, a melt-curve analysis was performed immediately after the amplification protocol.

### 4.5. Transfection of miRNA Mimics and miRNA Inhibitor

The mir-205 oligonucleotides (mimics, inhibitors and negative control) were designed and purchased by GenePharma (GenePharma Co., Ltd., Shanghai, China). All oligonucleotides were dissolved in diethylpyrocarbonate (DEPC)-treated water. Subconfluent proliferating cells were transfected with Lipofectamine 2000 (Invitrogen, Carlsbad, CA, USA), according to the manufacturer’s protocols. The cells were harvested 48 h after transfection. The final concentration of mimics, inhibitors or negative control was 50 nM. For long-term detection, mimics, inhibitors and negative control were repeatedly transfected every 3 days.

### 4.6. Plasmid Construction of SATB2 Over-Expression Vectors

The full-length open reading frame (ORF) of SATB2 was cloned into pEGFP-N1 vectors to generate SATB2 over-expression vectors. A high purity plasmid rapid extraction kit (QIAgen Biotech Co., Ltd., Germantown, MD, USA) was used to extract and amplify the plasmid. After sequencing, the plasmid solution was kept at −20 °C. Next, the complexes of plasmid and liposome were prepared according to recommended preparation methods of Lipofectamine 2000 Reagent Kit (Invitrogen, Carlsbad, CA, USA). Site-directed mutagenesis of the miR-205 seed sequence in the 3'-UTR (Mut) was performed using the QuikChange™ Site-Directed Mutagenesis Kit (Stratagene, La Jolla, CA, USA).

### 4.7. Bioinformatics Analysis

To identify the target genes of mir-205, we searched for candidate genes using three miRNA target prediction databases: TargetScan (www.targetscan.org/), miRanda (www.microrna.org/) and miRWalk (http://www.umm.uni-heidelberg.de/apps/zmf/mirwalk/index.html).

### 4.8. Luciferase Assays

For luciferase assays, BMSCs were cultured in 12-well plates and co-transfected with luciferase reporter plasmids, miR-205 mimics and pEGFP-N1 vectors using Lipofectamine 2000 (Invitrogen, Carlsbad, CA, USA). Cells were harvested and lysed 48 h after transfection, and luciferase activity was measured using the Dual-Luciferase Reporter Assay System (Promega, Madison, WI, USA). Renilla-luciferase was used for normalization. The experiments were performed independently in triplicate.

### 4.9. Alizarin Red S Staining

The formation of mineralized matrix was determined by Alizarin Red S staining. BMSCs with DM were washed twice with PBS and fixed with 70% ethanol for 1 h at room temperature, then stained with 40 mM Alizarin Red S for 10 min. Cells were washed with PBS twice and the stained matrix was photographed with an Olympus digital camera. Five images were taken and analyzed for total number with an Image Pro plus 6.0 software (Media Cybernetics, Rockville, MD, USA). For quantification of staining, the Alizarin Red S staining was released from the cell matrix by incubation in cetyl-pyridinium chloride for 15 min and the amount of released dye was measured by spectrophotometry at 540 nm. The results were normalized to total cellular protein content.

### 4.10. Western Blot Analysis

For western blot analysis, cell lysates were prepared by incubation on ice with lysis buffer (50 mM Tris-HCl (pH 7.5), 20 mM NaCl, 5 mM EDTA, 1% TX-100, 0.1% SDS, 5% glycerol + protease inhibitors), and centrifuged at 20,000× *g*. The supernatant was collected and protein concentration was determined using the Pierce BCA Protein Assay Kit (Thermo, Waltham, MA, USA) with bovine serum albumin as a standard control. The supernatant was mixed with equal volume of sample buffer (62.5 mM Tris, pH 6.8, 2% SDS, 5% mercaptoethanol, 1% bromophenol blue, and 25% glycerol). Then the mixture was boiled for 5 min, and centrifuged for 10 min at 10,000× *g*. Protein extractions were separated by using SDS-PAGE on 10% polyacrylamide gels, and transferred to polyvinylidene difluoride (PVDF) membranes (Millipore, Billerica, MA, USA). After blocking for 1 h with 8% skimmed milk in TBS buffer (10 mM Tris, 150 mM NaCl), the membrane was incubated with primary antibodies, including rabbit anti-BSP (1:500 dilution, Abcam, Cambridge, UK), rabbit anti-OPN (1:500 dilution, Abcam), rabbit anti-STAB2 (1:200 dilution, Santa Cruz Biotechnology, Santa Cruz, CA, USA), rabbit anti-Runx2 (1:400 dilution, Santa Cruz Biotechnology), rabbit anti-p-p38 MAPK (1:500 dilution, Santa Cruz Biotechnology), rabbit anti-p38 MAPK (1:1000 dilution, Santa Cruz Biotechnology), rabbit anti-p-ERK (1:1000 dilution, Santa Cruz Biotechnology), rabbit anti-ERK (1:1000 dilution, Santa Cruz Biotechnology) and mouse anti-β-actin monoclonal antibody (1:1000 dilution, Santa Cruz Biotechnology), overnight at 4 °C. Membranes were then washed and incubated with the appropriate secondary antibodies and detected the immunoactive signal with an ECL-based FluorChem^®^ FC2 image system (Alpha Innotech, San Jose, CA, USA). The FluorChem^®^ FC2 software was used to analyze the gray value of the protein expression in each group.

### 4.11. Statistical Analysis

All experiments were performed at least three times, and results were expressed as the means ± standard derivation (SD). The results were analyzed by one-way ANOVA followed by the SNK-q test for multiple comparisons. All analyses were performed using the Statistical Package for the Social Sciences (SPSS 13.0) software (SPSS, Chicago, IL, USA). Data were considered statistically significant at a *p* value <0.05.
